# Induction of Filopodia During Cytomegalovirus Entry Into Human Iris Stromal Cells

**DOI:** 10.3389/fmicb.2022.834927

**Published:** 2022-04-05

**Authors:** Kenneth Chang, Hardik Majmudar, Ritesh Tandon, Michael V. Volin, Vaibhav Tiwari

**Affiliations:** ^1^Department of Microbiology and Immunology, College of Graduate Studies, Chicago College of Osteopathic Medicine, and Chicago College of Pharmacy, Midwestern University, Downers Grove, IL, United States; ^2^Department of Microbiology and Immunology, University of Mississippi Medical Center, Jackson, MS, United States

**Keywords:** heparan sulfate, virus entry, 3-*O* sulfated heparan sulfate, virus cell to cell spread, actin cytoskeletal network

## Abstract

Many viruses exploit thin projections of filopodia for cell entry and cell-to-cell spread. Using primary cultures of human iris stromal (HIS) cells derived from human eye donors, we report a significant increase in filopodia formation during human cytomegalovirus (HCMV) infection. Using confocal microscopy, we observed a large number of virions being frequently associated along the filopodia prior to cell infection. Depolymerization of actin filaments resulted in a significant inhibition of HCMV entry into HIS cell. Our results further revealed that the transient expression of HCMV envelope glycoprotein B (gB) triggers the induction of the filopodial system. Since gB is known to bind the diverse chains of heparan sulfate (HS), a comparative study was performed to evaluate the gB-mediated filopodial induction in cells expressing either wild-type HS and/or 3-O sulfated HS (3-*O*S HS). We found that cells co-expressing HCMV gB together with the 3-O sulfotranseferase-3 (3-*O*ST-3) enzyme had a much higher and robust filopodia induction compared to cells co-expressing gB with wild-type HS. The above results were further verified by pre-treating HIS cells with anti-3-*O*S HS (G2) peptide and/or heparinase-I before challenging with HCMV infection, which resulted in a significant loss in the filopodial counts as well as decreased viral infectivity. Taken together, our findings highlight that HCMV entry into HIS cells actively modulates the actin cytoskeleton *via* coordinated actions possibly between gB and the 3-*O*S HS receptor to influence viral infectivity.

## Introduction

The tight bundles of filamentous actin in filopodia and in other cellular extensions such as lamellipodium are highly dynamic structures that facilitate infection of a wide variety of viruses at multiple steps in their life cycle (Gruenheid and Finlay, 2003; Akhtar and Shukla, 2009; Chang et al., 2016; Miranda-Saksena et al., 2018). This includes the reorganization of the membrane cytoskeleton in the formation of filopodia during the initial virus–cell contact which not only generates a large surface area for incoming virions but also promotes viral gliding or surfing along filopodia. This allows the virus to attach to the maximum number of host cells even without reaching the cell body (Schudt et al., 2013; Chang et al., 2016; Mehedi et al., 2016; Marzook and Newsome, 2017). Some viruses use an endocytic mode of viral entry in which virus-internalization receptor complexes are formed in filopodia to promote virus internalization and trafficking (Apodaca, 2001; Lehmann et al., 2005; Mercer and Helenius, 2008; Mothes et al., 2010; Oh et al., 2010). Interestingly, human respiratory syncytial virus (RSV) infection of lung cells upregulates filopodia formation and increases cell motility using host cell actin-related protein 2 (ARP2) (Mehedi et al., 2016). Uniquely, filopodia also facilitate RSV spread by shuttling the virus to nearby uninfected cells (Mehedi et al., 2016). Additionally, filipodia are well documented in mediating viral egress and budding (Kolesnikova et al., 2007; Carpenter et al., 2008; Lu et al., 2013; Chang et al., 2016; Gordon et al., 2019).

Since filopodia are considered as specialized sensory protrusions with the ability to probe their nearby microenvironment (Jacquemet et al., 2019), it is not a big surprise that innate immune cells such as macrophages and neutrophils are also equipped with filopodia which they use to patrol for pathogens and, paradoxically in some cases, end up spreading viral infections *via* these membrane processes (Möller et al., 2013; Hoffmann et al., 2014; Stow and Condon, 2016). Along similar lines, virtually every cell releases nanovesicles in the form of exosomes which act as intercellular messengers for transferring proteins, lipids, and genomic cargo. This process can serve as a regulator of both the innate and adaptive immune systems by stimulating cytokine production, inflammatory responses, and antigen presentation (Abels and Breakefield, 2016; Carriere et al., 2016). Interestingly, vesicular exosomes not only surf on filopodia before their internalization in endocytic vesicles (Heusermann et al., 2016; Schneider and Simons, 2016) but also play a significant role in viral pathogenesis by altering host defense mechanisms and facilitating dissemination of the microbes (Wiley and Gummuluru, 2006; Yao et al., 2018).

Growing evidence further indicates that filopodial extensions are stimulated during host cell injury, as a result of damage associated with inflammation and during angiogenic processes (Owen et al., 2007; Schweer et al., 2013; Stow and Condon, 2016). In this regard, active sprouting of filopodia in both leukocytes and endothelial cells has been reported, which links their role in events impacting disease development (Do et al., 2014; Quick et al., 2014; Chang et al., 2016; Kim et al., 2017; Kroon et al., 2018). Therefore, filopodia formation is a potential target for drug development, as preventing filopodia formation may affect viral binding, surfing, and internalization. Since viruses are also dependent on the host cell cytoskeleton during their egress process, additional new avenues are likely to be developed including post-viral entry inhibitors targeting virus migration or dissemination. Similar approaches are already being developed against various forms of cancer (Kitagawa et al., 2010; Zins et al., 2013; Castillo-Pichardo et al., 2014; Becker et al., 2016).

In this study, we used our previously established human iris stroma (HIS) cell culture model (Baldwin et al., 2013; Baldwin et al., 2015) to investigate the role of filopodia during human cytomegalovirus (HCMV) infection. Our findings demonstrate that HIS cells challenged with HCMV induce filopodia formation as early as 60 min post-infection (p.i.) and remain visible for up to 10 h p.i. Upon the use of an inhibitor to actin polymerization (cytochalasin D), a significant inhibition in filopodia and HCMV entry was noticed. Further, we found that the transient expression of HCMV glycoprotein B (gB) induces the filopodia formation, which suggests a novel involvement of gB in cellular remodeling. Since HCMV gB is a viral ligand known to recognize the heparan sulfate (HS) receptor (Shukla and Spear, 2001; Isaacson and Compton, 2009), we compared the interaction between gB and the wild-type HS to the interaction between gB and the modified form of HS (3-O sulfated HS) on their induction of filopodia. Our results indicate that the co-expression of HCMV gB together with 3-*O*S HS resulted in a robust induction of filopodia compared to the cells co-expressing HCMV gB with wild-type HS. This suggests that the presence of sulfation is likely one of the critical factors for the enhanced filapodial system. This result was further supported by findings where the masking of 3-O sulfation by anti-3-*O*S HS (G2) peptide (Tiwari et al., 2011) and/or by removal of HS including 3-*O*S HS by heparinase I impaired the filopodial count and the HCMV infectivity. Thus, our study highlights the critical priming event in which the initial viral ligand (HCMV gB) and host cell receptor (3-*O*S HS) interactions likely generate the filopodial system resulting in efficient viral infectivity.

## Materials and Methods

### Cell Culture, Virus, Antibody, and Chemicals

As described previously, the HIS cultures were prepared in accordance with institutional review board-approved protocols and were isolated from anonymously donated human eyes (provided by the Illinois Eye Bank, Chicago, IL, United States) *via* sterile dissection of the iris and removal of the pigmented epithelial layer with a sterile cotton swab (Baldwin et al., 2013). The tissue was then digested with 0.2% type II collagenase (Sigma-Aldrich, St. Louis, MO, United States) in cell culture medium, MCDB-131 (Sigma-Aldrich, St. Louis, MO, United States) at 37°C with gentle stirring for 20–30 min. Digested tissues were next centrifuged to remove tissue debris, and HIS cells were cultured in MCDB-131 containing 10% fetal bovine serum (FBS) and antibiotics. Wild-type Chinese hamster ovarian (CHO-K1) cells were grown in Ham’s F12 (Ham’s F-12 medium (Gibco/BRL, Carlsbad, CA, United States) supplemented with 10% FBS and streptomycin/penicillin (P/S) (Gibco/BRL) (Clement et al., 2006), while HIS cells were cultured in MCDB-131 containing 10% FBS and antibiotics (Baldwin et al., 2013). The merlin strain of human herpesvirus 5 (ATCC^®^ VR-1590 TM) used in this study was purchased from ATCC. HCMV was detected using a fluorescein isothiocyanate (FITC)-conjugated antibody directed against HCMV envelop gB (Virostat, Inc.). Cytochalasin D and heparinase-I were obtained from Sigma-Aldrich.

### Plasmid and Cell Transfection

Mammalian expression plasmid encoding human 3-*O*ST-3B1 (H3ST3B1), empty vector (pCDNA3.1), and expression plasmid encoding HCMV gB tagged with pDS-Red were used in this study. 3-O sulfotranseferase-3 (3-*O*ST-3) and HCMV gB expression plasmids were kindly provided by Professor Deepak Shukla (University of Illinois at Chicago) and Dr. Tandon (University of Mississippi Medical Center). CHO-K1 and HIS cells were transfected with control vector pCDNA3.1, 3-*O*ST-3, and HCMV gB expressing plasmids using Lipofectamine 2000 (Invitrogen) as previously described (Baldwin et al., 2015).

### Immunofluorescence and Deconvolution Microscopy

Target HIS cells were challenged with merlin strain of HCMV (37°C at 5% CO_2_) at 10 multiplicity of infection (MOI) on different time points (60 min, 5, and 10 h). Immunofluorescence staining was performed to detect HCMV using a FITC-conjugated antibody directed against HCMV envelop gB (Virostat, Inc.) at different time points as previously described (Baldwin et al., 2015). In a separate excrement, HIS cells were pre-treated with anti-3-*O*S HS (G2) peptide at 1 mM and/or control peptide (Cp) (Tiwari et al., 2011) for 2 h before challenging with HCMV infection for 60 min followed by immunofluorescence staining for HCMV using anti-HCMV gB antibody. In a parallel experiment, HIS cells were also pre-treated with heparinase-I (1.0 U/ml) and/or cytochalasin D (1.0 μg/ml) for 1 h before challenging with HCMV (10 MOI) infection for 2 h followed by immunofluorescence staining for HCMV using anti-HCMV gB antibody. The infection was performed in serum-free media, OptiMEM (Invitrogen), followed by fixation of cells p.i. using fixative buffer (2% formaldehyde and 0.2% glutaraldehyde). The cells were then washed and permeabilized with buffer containing 2 mm MgCl_2_, 0.01% deoxycholate, and 0.02% Nonidet NP-40 for 20 min. After rinsing, 10 nM rhodamine-conjugated phalloidin (Invitrogen) was added for F-actin staining at room temperature for 45 min. In a parallel experiment, CHO-K1 cells were transfected with pDS-Red plasmid encoding an empty vector (pCDNA3.1) and/or HCMV gB-pDS-Red or together with 3-*O*ST-3. The DNA concentrations were balanced and kept equal to 2.5 μg throughout the experiments. The cells were washed with 1 × phosphate-buffered saline (PBS), fixed, and permeablized followed by staining using phalloidin dye (Alexa fluor^®^ 488; Invitrogen) for visualizing filopodia. Finally, the cells were washed; the images of labeled cells were acquired using a confocal microscope (Nikon A1R), and filopodial extensions were analyzed (metamorph). The images of HCMV infected HIS cells were deconvolved at × 40 magnification with NIS-Elements AR software. Orthogonal sections were made in a z stack of 25 images (0.5-μm interval). A midsection of an individual stack taken as a part of z series was shown for maximum light intensity. The picture was produced in Adobe Photoshop 7.0.

### Quantification of Cellular Protrusions

All cellular protrusions (filopodia and lamellipodia) and stress fibers were measured on 15 randomly selected fixed HIS and/or CHO-K1 cells within the field of view and an observer blinded in triplicate experiments. Protrusions were scored based on their morphology. Protrusions that ranged from 1 to 10 μm without a visible head were counted as filopodia. Protrusions were counted for each cell and then averaged for the 15 observed cells in triplicate experiments. The length of the longest filopodia was measured using a standardized ruler (image-stamped on each image) by the ImageJ program; by visual exam, the length of the filopodia was approximated in relation to the ruler. Protrusions with a bulbous head wider than its base were counted as lamellipodia. Then, the number of protrusions was averaged for the 15 observed cells in the triplicate experiment. Linear striations through each cell were counted as stress fibers. Using a scale ranging from 0 to 3 (with 0 being no stress fibers and 3 being many stress fibers), each cell was scored between 0 and 3. The stress fiber score was then averaged for the 15 cells in the triplicate experiment. Two researchers performed all the measurements independently. All statistical analysis was done using XLSTAT add-in for Microsoft Excel (Addinsoft, NY, United States) or Student’s *t*-test (Microsoft Excel).

### Human Cytomegalovirus gB Expression Analysis Using Cell ELISA

CHO-K1 cells were grown to 70–90% confluence followed by cell transfection using HCMV gB plasmid (pCCMVgB) or in parallel with pCDNA at 2.5 μg DNA using Lipofectamine 2000 (Thermo Fisher Scientific, Waltham, MA, United States) with overnight incubation. The cells were then washed in Tris-buffered saline (TBS), fixed with methanol for 5 min, and washed again. Cell ELISA protocol described previously was then followed using 0.5 μg/ml monoclonal antibody to HCMV gB (Acris, Rockville, MD, United States, cat. no. BM3261) and goat anti-mouse peroxidase-conjugated secondary antibody diluted 1:10,000 (Thermo Fisher Scientific, Waltham, MA, United States). Final washing of the plate was done three times in Tris-buffered saline Tween-20 (TBST) before 3,3′,5,5′-tetramethylbenzidine (TMB) substrate (Pierce Biotechnology, Rockford, IL, United States) was added and the reaction was stopped. The enzymatic activity was measured at OD 450 nm by a microplate photometer (Thermo Scientific Multiskan FC).

### Confirmation of 3-*O*ST-3 Expression Using Herpes Simplex Virus Reporter-Based Entry Assay

CHO-K1 cells were grown in six-well plates to subconfluence and transfected with 2.5 μg of human 3-*O*ST isoforms (3-*O*ST-3) or control plasmid (pDream2.1 or pCDNA3.1) using Lipofectamine 2000 (Thermo Fisher Scientific, Waltham, MA, United States). The soup for transfection came from the cells in parallel transfected for imaging experiment involving human 3-*O*ST-3. At 16 h post-transfection, the cells were replated into 96-well dishes followed by infection with β-galactosidase expressing recombinant HSV-1 gL86 virus. After 6-h p.i., β-galactosidase assay was performed using a soluble substrate o-nitrophenyl-β-D-galactopyranoside (ONPG; ImmunoPure, Pierce). The enzymatic activity was measured at 410 nm using a microplate reader.

## Results and Discussion

### Induction of Filopodia During Human Cytomegalovirus Entry Into Human Iris Stromal Cells

**FIGURE 1:**
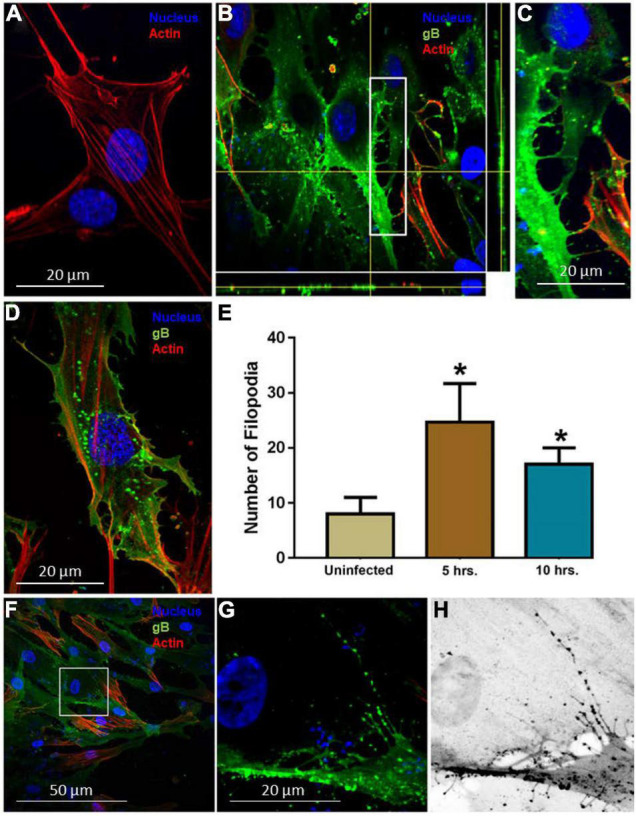
Induction of filopodia in human iris stromal (HIS) cells upon HCMV infection. **(A)** Mock infected HIS cells. **(B,C)** HIS cells infected with HCMV at 10 multiplicity of infection (MOI) for 5 h. Immunofluorescence staining involving fluorescein isothiocyanate (FITC)-conjugated anti-gB antibody showed the presence of the virus inside the HIS cells using deconvolution imaging. The area boxed in **(B)** is highlighted in **(C)** showing a large number of virus particles at the cell body beneath filopodia. The cells were stained with phalloidin conjugated to rhodamine to detect filopodia, while nuclei were stained with 4,6-diamino-2-phenylindole (DAPI). **(D)** HIS cells infected with HCMV at 10 MOI for 10 h. At this time point, a large number of virus particles were localized near the nucleus, but filopodial protrusions remained visible. **(E)** Quantification of filopodia and its length were based on 15 cells randomly picked per triplicate experiment. Asterisks indicate significant difference from controls and/or treatments (*p* < 0.05, *t*-test), and error bars represent SD. **(F–H)** Immunofluorescence imaging performed 60 min post-HCMV infection by using anti-HCMV glycoprotein B (gB) antibody in HIS cells. The area boxed in **(F)** is highlighted in **(G)**, and inverted gray scale image is projected in **(H)**. The presence of green punctate indicates HCMV particles present on cellular protrusions. Imaging was performed by using Nikon microscope at ×40 objective and Nikon software.

To establish the HCMV entry model using HIS cell, the cells were challenged with human HCMV, while mock-infected HIS cells were used as a control. The mock-treated and virus-infected cells were fixed and treated with FITC-conjugated antibody directed against the HCMV envelop gB (Virostat Inc.) to detect HCMV. The rhodamine phalloidin dye was used to stain the actin cytoskeleton. As indicated in Figure 1A, mock-infected HIS cells had a smooth surface with few stress fibers and filopodia. In contrast, HIS cells challenged with HCMV showed a significant amount of filopodial protrusions (Figures 1B–H). At 5 h p.i., the majority of virions were found inside the cells as evident from the deconvolution stacking (Figure 1B). Further, a closer visual look showed that the virus punctate were mostly present at the base of filopodia (Figure 1C). In contrast, in HIS cells infected for 10 h, the virus presence was mostly visible close to the nucleus (Figure 1D); however, the filopodia remained visible. Since the induction of filopodia during viral entry has been documented as early as 30 min p.i. (Clement et al., 2006; Dixit et al., 2008; Jessica et al., 2008; Xu et al., 2015), we decided to investigate early stages of HCMV entry in HIS cells. HIS cells were challenged with HCMV at 10 MOI for 60 min and then examined by fluorescence microscopy. As indicated in Figures 1F–H, large numbers of HCMV particles were frequently observed aligned in a chain on the abundant filopodia suggesting the possibility of viral surfing—a phenomenon which is shared by multiple viruses including herpes simplex virus (Lehmann et al., 2005; Burckhardt and Greber, 2009; Oh et al., 2010). The number of filopodia was also quantified in triplicate experiments and was significantly higher in the infected cells (Figure 1E). Interestingly, a significant increase in actin stress fibers was also observed in HIS cells infected with HCMV, compared to mock-infected cells (data not shown). To rule out the significance of filopodia during HCMV entry into HIS cells, the cells were pre-treated with the actin depolymerizing agent Cytochalasin-D (cyto-D) before challenging them with the HCMV. The outcome of this experiment clearly showed a drastic reduction in the virus infectivity upon confocal imaging (data presented as Supplementary Figure 1). Future studies involving multiple HCMV dosage under different time points will be helpful to generate the kinetics of filopodial development during HCMV entry.

### Expression of Human Cytomegalovirus Glycoprotein B Induces Actin Cytoskeletal Changes and Filopodia Formation

**FIGURE 2:**
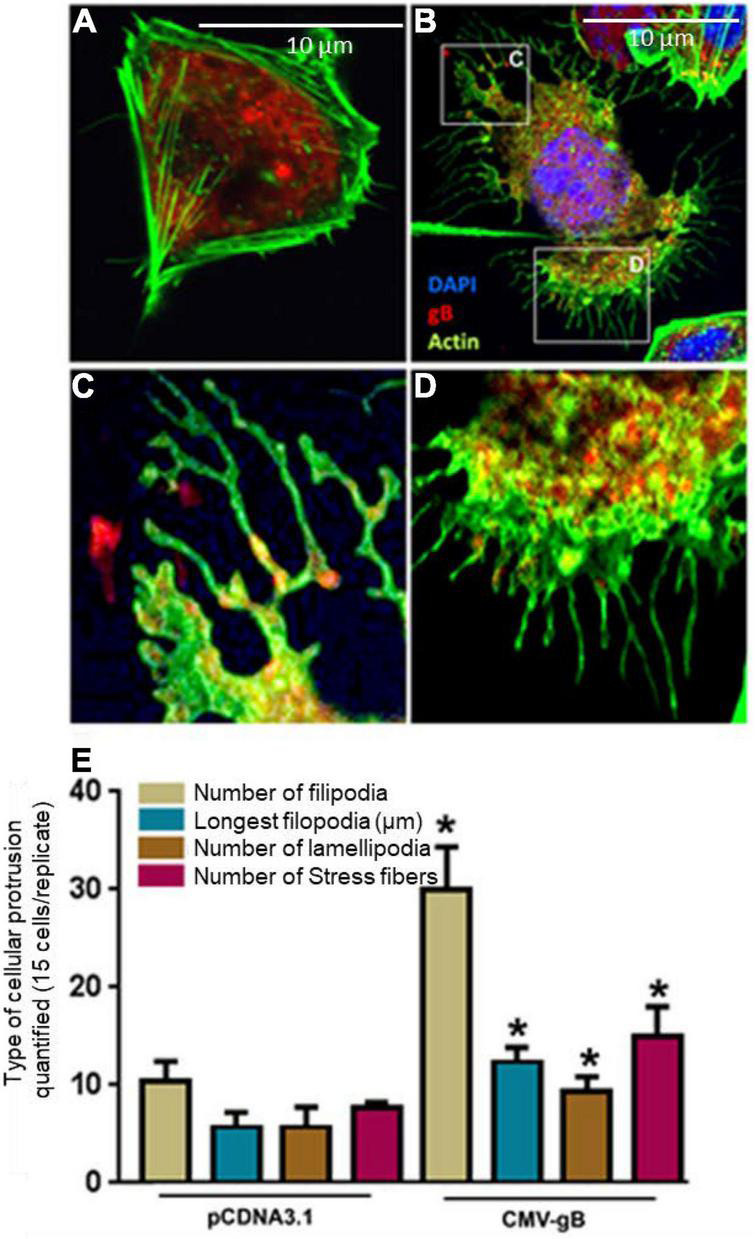
Expression of HCMV glycoprotein B (gB) leads to the induction of cellular protrusions in Chinese hamster ovary (CHO-K1) cells. **(A)** CHO-K1 cells transfected with pDS-Red encoding pCDNA3.1 (2.0 μg) plasmid using lipofecatamine-2000. Cellular actin was stained using (Alexa fluor^®^ 488; Invitrogen) for visualizing filopodia. **(B)** CHO-K1 cells transfected with 2.0 μg HCMV gB tagged with pDS-Red. The boxed region in **(B)** is highlighted in **(C,D)**. **(C)** Showed the presence of HCMV gB on the tips of the filopodia, while **(D)** shows extensive gB clustering inside cell at the base of filopodia. **(E)** Quantification of cellular protrusions (filopodia, length, lamellipodia, and stress fibers) is being compared between CHO-K1 cells expressing pCDNA3.1 and CHO-K1 cells expressing HCMV gB. The quantifications are based on 15 cells randomly picked per triplicate experiment. Asterisks indicate significant difference from controls and/or treatments (*p* < 0.05, *t*-test), and error bars represent SD.

Since HCMV gB is known to interact with cell surface HS together with the fact that the uniquely sulfated and unsulfated moieties in HS chain (3-*O*S HS) are expressed on filopodia (Oh et al., 2010; Whittall et al., 2013; Sharthiya et al., 2017), we decided to quantify the filopodial growth between the cells expressing wild-type HS or 3-*O*S HS when co-expressed with HCMV gB. In this experiment, we preferentially selected Chinese hamster ovary (CHO-K1) cells over HIS cell, since CHO-K1 cells naturally express HS (Bame, 1993) but lack the endogenous 3-*O*ST-3 enzyme (Shukla et al., 1999), while in contrast, HIS cells express both HS and 3-*O*S HS (Baldwin et al., 2013). Therefore, CHO-K1 cell provided an ideal platform to test the selective interactions between gB with HS and/or gB with 3-*O*S HS. The CHO-K1 cells were transfected with gB-tagged with pDS-Red plasmid, while in parallel control CHO-K1 cells were transfected with pDS-Red-tagged pCDNA3.1 plasmid using lipofectamine as previous described (Baldwin et al., 2013). As indicated in Figure 2A, wild-type HS CHO-K1 cells expressing an empty vector had very few protrusions compared to CHO-K1 cells expressing the wild-type HS and HCMV gB (Figure 2B). Interestingly, HCMV gB (red) localization was observed both at the tip of filopodia (Figure 2C) and at the base of filopodia in clusters (Figure 2D). Further, upon quantification, the number of filopodia, their length, number of lamellipodia, and the number of stress fibers were all significantly higher in CHO-K1 cells expressing HCMV gB compared to control cells expressing empty vector (Figure 2E). These findings highlight a new role for HCMV gB in promoting filopodial extensions which suggests the possibility that incoming HCMV virions carrying multiple copies of gB may facilitate the actin-rich protrusions of filopodia. The later process may aid in viral surfing and bringing the virus to the base of filopodia where endocytic trafficking receptors are primed for viral internalization. Interestingly, envelop F protein of human RSV has recently been shown to induce filopodia (Mehedi et al., 2016), while in the case of Alphavirus a replicase protein, NSP1, has been shown to induce filopodia formation (Laakkonen et al., 1998). Similarly, a Negative Regulatory Factor (Nef) protein in human immunodeficiency virus (HIV) is known to induce actin cytoskeleton changes impairing cell migration toward chemokines (Nobile et al., 2010). Our previous studies involving cultures of corneal stroma, retinal pigmented epithelial cells, and the cells of neuronal origin showed induction of cellular protrusions upon viral infection (Clement et al., 2006; Dixit et al., 2008; Tiwari et al., 2008). In fact, a recent study with SARS CoV-2 suggested that the actin cytoskeleton is required during HS-assisted viral entry (Zhang et al., 2020). On a similar note, the disruption of the host cytoskeleton homeostasis has been linked to coronavirus-associated pathology such as defective cytokinesis, demyelination, cilia loss, and neuron necrosis (Wen et al., 2020).

### Co-expression of Human Cytomegalovirus-gB Together With Heparan Sulfate Modifying Enzyme 3-*O*ST-3 Efficiently Enhances the Induction of Filopodia

**FIGURE 3:**
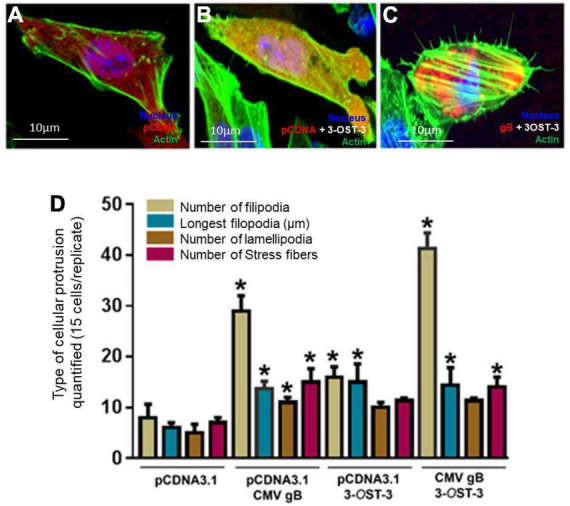
Co-expression of HCMV glycoprotein B (gB) together with 3-*O* sulfated heparan sulfate (3-*O*S HS) efficiently enhances the cellular filopodial protrusions in the CHO-K1 cell model. Several combinations were used in this experiment to image and quantify filopodia. Individual CHO-K1 cells were separately transfected with pDsRed encoding pCDNA3.1 plasmid **(A)**, plasmid encoding 3-*O*ST-3 enzyme alone **(B)**, and co-transfected with HCMV-gB together with 3-*O*ST-3 plasmid **(C)**. The DNA concentration was kept constant throughout the experiment. The cells were fixed 12 h post-transfection and imaged for quantification of cellular protrusions. **(D)** Quantification of cellular protrusions (filopodia, length, lamellipodia and stress fibers) is being compared between CHO-K1 cells expressing pCDNA3.1, HCMV gB, 3-*O*ST-3, and 3-*O*ST-3 along with HCMV gB. The quantification is based on 15 cells randomly picked per triplicate experiment. Asterisks indicate significant difference from controls and/or treatments (*p* < 0.05, *t*-test), and error bars represent SD.

We next investigated whether co-expression of HCMV-gB together with 3-*O*S HS receptor will further impact filopodial induction. The logical rationale to test this hypothesis was based on the fact that the sulfation in HS chain generates multi-ligand biding sites which could also display a higher binding affinity for HCMV gB. In addition, our previous finding had also shown that HS and 3-*O*S HS receptors are expressed on cellular protrusions enhancing viral infectivity (Clement et al., 2006; Oh et al., 2010). Similarly, our earlier study also indicated that effector cell expressing HCMV-glycoproteins including gB preferentially show a higher affinity for 3-*O*S HS receptor over wild-type HS to promote cell-to-cell fusion (Baldwin et al., 2015; Sharthiya et al., 2017). Therefore, we decided to use Chinese hamster ovary (CHO-K1) cells in co-expression experiments. Although historically, CHO-K1 cells have been widely used in the discovery of viral entry receptors (Shukla et al., 1999), we chose them in our experiments because they lack the endogenous expression of 3-O sulfotransferases (3-*O*STs) enzymes to generate 3-O sulfated HS (Shukla et al., 1999). In addition, CHO-K1 cells are easy to transfect and subculture, and therefore they provide an excellent platform to study the role of 3-*O*STs in HCMV entry. In this experiment, CHO-K1 cells were co-transfected with mammalian expression plasmid encoding 3-*O*ST-3 along with pDS-Red tagged HCMV gB. In parallel, CHO-K1 cells transfected with DsRed-pCDNA3.1 and/or 3-*O*ST-3 alone served as a control. The DNA concentrations were balanced and used at 2.0 μg for the tested combinations. The cells were fixed using fixative buffer 12 h post-transfection followed by staining using Alexa fluor^®^ 488 phalloidin dye (green) for imaging and filopodia quantification. As indicated in Figure 3A, wild-type HS expressing CHO-K1 cells transfected with pCNDA3.1 alone had very little visible filopodia, while filopodial extensions were frequently observed in CHO-K1 cells expressing 3-*O*ST-3 which were mostly localized on cellular tips and as filopodial bridges (Figure 3B). However, CHO-K1 cells expressing HCMV gB together with 3-*O*ST-3 generated the greatest number of filopodia which were visible on the cell surfaces (Figure 3C). Upon quantification, it was clear that combined expression of both HCMV gB and 3-*O*S HS stimulated the maximum cellular protrusions of filopodia compared to the cells expressing HCMV gB alone (Figure 3D). Expression of 3-*O*ST-3 was confirmed using the transfection supernatant obtained from the above experiment using CHO-K1 cells followed by running an ONPG-based colorimetric assay after exposing the cells with the reporter HSV-1 (Supplementary Figure 2), while the expression of HCMV gB was also confirmed using cell ELISA (Supplementary Figure 3).

### Pre-treatment of Human Iris Stromal Cells With Anti-3-*O*S Heparan Sulfate (G2) Peptide and/or With Heparinase-I Inhibits Filopodia Formation and the Virus Localization Along the Filopodia

**FIGURE 4:**
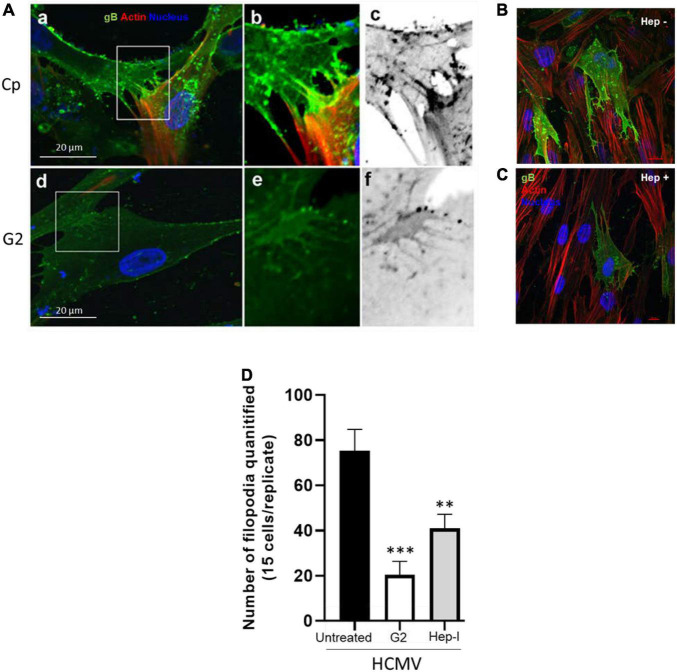
Pre-treatment of HIS cells either with the anti-3-*O*S HS (G2) peptide and/or with heparinase-I interferes with the induction of filopodia and HCMV infectivity. **(A)** HIS cells pre-treated with control peptide (Cp) (a–c) and/or pre-treated with anti-3-*O*S HS (G2) peptide (d,e) for 2 h followed by HCMV infection at 10 MOI for 60 min. The cells were washed and fixed for imaging HCMV infection and filopodia induction. The cells pre-treated with Cp had a large number of viruses on the filopodial bridges between cells (a–c). In contrast, cells treated with G2 peptide had significantly a smaller number of filopodia and fewer viruses on HIS cells (d,e). **(B)** In this experiment, HIS cells were pre-treated with heparinase-I at 1.0 U/ml **(C)** and/or mock-treated (PBS) for 1 h followed by HCMV (10 MOI) infection for 2 h. The cells were then washed and fixed with FITC conjugated anti-gB antibody and phalloidin staining to detect virus and the filopodial growth using Nikon A1R confocal imaging at ×40. **(D)** The quantification of cellular filopodia for each group (mock treated; G2 peptide, and heparinase-I treated) is based on 15 cells randomly picked per triplicate experiment. Asterisks indicate significant difference from controls and/or treatments (*p* < 0.005, one-way ANOVA followed by *post hoc* Bonferroni test) and error bars represent SD.

Finally, we rationalize that direct targeting of the 3-O sulfated moieties would prevent the critical ligand (gB) access to the receptor (HS and/or 3-*O*S HS) and hence may affect the filopodial induction and the HCMV infectivity. Therefore, in this experiment, we used HCMV susceptible HIS cells which naturally express 3-*O*S HS (Baldwin et al., 2013). In addition, we also took the advantage of our previously characterized phage display derived G2 peptide which specifically recognizes regions of 3-*O*S HS in HS chain and is a proven anti-HCMV molecule (Tiwari et al., 2011; Dogra et al., 2015). Briefly, HIS cells were pre-treated with G2 (MPRRRRIRRRQK) peptide at 1 mM concentration, while in parallel HIS cells were also pre-treated with the Cp (RVCGSIGKEVLG). After 2 h of pre-treatment, the cells were challenged with HCMV at 10 MOI for 60 min. The cells were then washed three times with 1 × PBS to remove the unbound virus and fixed followed by staining using anti-HCMV gB antibody. As shown in Figures 4Aa–c, a larger number of filopodial growth, especially at cell-to-cell connections, were observed in Cp-treated cells infected with HCMV. In contrast, G2 peptide-treated cells had lower filopodia and virions at cell-to-cell junctions (Figures 4Ad–f). Similarly, the images generated for the HIS cells pre-treated with heparinase-I showed inhibition in both the filapodial counts and in the viral infectivity (Figures 4B–D). The above results demonstrate that loss of 3-*O*S HS receptor either from the cell surface or from the filopodia affects the entry of HCMV into HIS cells. In fact, previous studies in endothelial cells have demonstrated the presence of 3-*O*S HS on filopodial structures (Whittall et al., 2013). Similarly, our previous findings have also shown that masking of 3-*O*S HS receptor *via* G2 peptide significantly blocks entry of HCMV in multiple other cell-types (Tiwari et al., 2011; Baldwin et al., 2015) including HIS cell.

## Discussion

**FIGURE 5:**
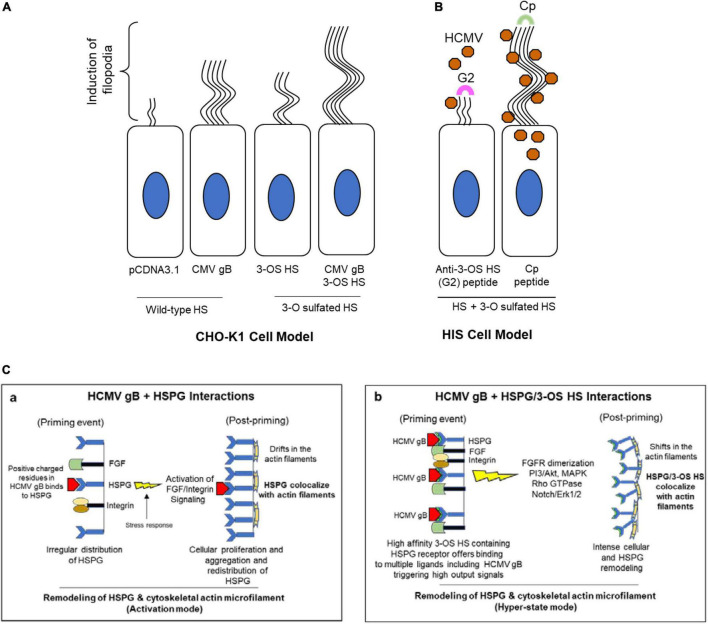
Induction of filopodial system under different conditions in HCMV entry models. **(A)** In CHO-K1 cell model, a higher number of filopodial extensions were noticed during co-expression of HCMV gB together with 3-*O*ST-3 enzyme. **(B)** In primary cultures of HIS cell model, the blocking of 3-O sulfation in HS chain by the pre-treatment of the cells with anti-3-*O*S HS (G2) peptide but not the control peptide (Cp) negatively impacts the filopodia including the virus infectivity. **(C)** Significance of ligand–receptor interactions as an important determinant for crosslinking the HSPG receptor along with the actin cytoskeleton for a remodeling event during HCMV entry. Essentially, HCMV gB interactions with wild-type HSPG likely leads to a productive actin and HSPG remodeling (a), while in the presence of a high-affinity 3-*O*S HS ligand, a hyper state remodeling event goes in effect (b) due to the multi-ligand–receptor interactions resulting from an intense cellular change including a high turnover in HSPGs.

Our study identified the induction of the filopodial system during HCMV entry into HIS cell. To demonstrate the significance of actin network in HCMV entry, the HIS cells were pre-treated with an actin depolymerizing drug which negatively impacted the HCMV infectivity (Supplementary Figure 1). Since the first step in viral entry involves the attachment step where HCMV gB recognizes a highly diverse chain of HS for the initial docking, we compared the response of gB to induce filopodia in the cell expressing wild-type HS and/or the cell expressing modified form of HS—3-O sulfated HS. We found that the target cell co-expressing HCMV gB together with 3-*O*ST-3 enzymes resulted in a much higher and robust filopodia induction compared to the cell co-expressing wild-type HS with gB (Figure 5A). In addition, upon blocking of 3-*O* sulfated HS by G2 peptide negatively impacted filapodial induction (Figure 5B). Our result that HCMV gB can induce filopodia with wild-type HS receptor was not surprising, since as per the classic ligand–receptor model, in the absence of ligand, heparan sulfate proteoglycan (HSPG) receptors are irregularly distributed on fibroblast cells (Martinho et al., 1996). In contrast, ligand binding (for example, lipoprotein lipase or antibodies against HS) to HSPG receptor results in clustering or aggregation of HSPG and in colocalization with the actin cytoskeleton (Martinho et al., 1996). Because HCMV gB is classified as a viral ligand for HS receptor (Compton et al., 1993), it is tempting to argue that a similar ligand (gB)–receptor (HSPG) interaction will generate a stress response triggering the FGF/integrins-mediated signaling along the actin cytoskeleton (Figure 5Ca). In fact, in the absence of gB, CHO-K1 cells expressing wild-type HS did not induce the filopodial system. On the other hand, a higher filapodial induction during gB interaction to 3-*O*S HS could be very well related to the unique ability of 3-O sulfation in generating multi-ligand biding sites (for example, HSV-1 glycoprotein D, antithrombin, neuropilin, FGF receptor, integrins), attracting a broad array of effectors which results in a robust signaling response compared to the wild-type HS for the filapodial induction. In fact, the most studied molecular mechanism of ligand–receptor complex formation and signaling activation mediated by HSPG has been linked to the sulfation pattern in HS. The later 3-O modification in HS transforms the overall HSPG profile as an intrinsic receptor to bind the FGF ligand and multiple other critical ligands resulting in activation of MAPK, PI3-akt/JAK/STAT, and Rho GTPase (De Pasquale and Pavone, 2020). Therefore, HCMV gB–3-*O*S HS interactions likely generate an exaggerated response for an intense HSPG remodeling (Figure 5Cb). To rule out the significance of 3-O sulfation in filapodial induction and virus infectivity, the HIS cells were either pre-treated with heparinase-I and/or by anti-3-*O*S HS (G2) peptide before HCMV infection. This result clearly showed the significance of 3-O sulfation in filopodial-mediated HCMV entry (Figures 4A,B). To rule out the differences in the signaling cascade and the associated outcome either during gB interaction to HS and/or gB interaction to 3-*O*S HS, additional experiments will be required. In this direction, we anticipate using phage display derived anti-HS antibodies targeting different regions in HS chain (van Kuppevelt et al., 1998). The latter will offer an ideal tool in dissecting the key residues in HS chain which are potentially involved in the filopodial development in HCMV entry.

In our CHO-K1 cell experiment gB and 3-*O*ST-3 were co-expressed in the same cell (cis) but not in trans. The information generated with CHO-K1 in the cis model is critical since in theory the virus surfing *via* filopodia would bring the gB (virions) and 3-*O*S HS into the same cell. However, the future investigation testing the interactions between gB 3-*O*S HS in trans will be worth investigating to better understand the molecular dynamics of the filopodial system. Similarly, investigating if other HCMV envelope glycoproteins such as glycoprotein M (gM) and N (gN) which are also known to bind HSPG (Carlson et al., 1997), is equally important in the filopodial expansion. Interestingly as gB is universally conserved among herpesviridae family (Isaacson and Compton, 2009), one interesting possibility would be to test if gB from the other herpes virus may share a similar phenotype to induce filopodia *via* their interaction with 3-*O*S HS receptor. Previous studies have already shown the presence of 3-*O*S HS on filopodial platforms (Whittall et al., 2013) including in HIS cells (Baldwin et al., 2015); therefore, it would also be a logical test if HCMV entry impacts HS biosynthesis pathways (NDST-1/EXT-1/2-O, 6-O, and 3-O sulfotransferases) and downstream in HSPG turnover. In fact, the studies conducted in the invasive cancer model do suggest an upregulation of 3-O sulfotransferase during reorganization of actin cytoskeleton (Denys and Allain, 2019). In our viral entry model, testing an upregulation in 3-*O*ST-3 enzyme in context with filopodial sprouting will also generate a significant interest regarding their potential role in HCMV-associated cancer (Richardson et al., 2020). Certainly, the presence of constantly emerging filopodia and/or filopodial bridges can be very handy for the virus as an efficient mechanism for the virus cell-to-cell transmission by avoiding immune surveillance (Zhong et al., 2013). The other striking feature of the filapodial system is to constantly evolve and expand even under the dense gel of the extracellular matrix which raises the importance of the involvement of endogenous heparanase in this process (Agelidis et al., 2017, 2021). The HSPG-mediated signaling favoring filopodial induction can be related to multiple pathways. For instance, activation of tyrosine kinase signaling (Ohkawa et al., 2021) by HSPG affects the actin rearrangements and the filopodia formation (Ren et al., 2019). In the case of herpes simplex virus (HSV-1), the Syndecan members of the proteoglycan family have been shown to play an important role in the induction of filopodia *via* Rho GTPases signaling which interestingly also affects the HS expression (Granés et al., 1999; Bacsa et al., 2011; Hadigal et al., 2020; Karasneh et al., 2021). Interestingly a recent study has shown that an adhesion molecule, CD44, which typically gets upregulated during cell assault and/or during inflammation, coats the filopodia with a hyaluronan-rich glycocalyx *via* ligand–receptor interactions (Härkönen et al., 2019). Interestingly, our finding can also be applicable to multiple other cell types since HCMV also exploits membrane ruffling during entry into dendritic cells (Haspot et al., 2012). Similarly, the HCMV-associated “filopodial system” together with actin-rich tunneling nanotubes can also be predicted to help virus migration to distant organs. In conclusion, a better understanding of the filopodial system especially in the clinical samples associated with HCMV (Hamouda et al., 2016; Rawlinson et al., 2016) may further provide the opportunities to identify novel molecular targets to improve therapeutic strategies.

## Data Availability Statement

The raw data supporting the conclusions of this article will be made available by the authors, without undue reservation.

## Author Contributions

KC and HM conducted the experiment and generated the data. VT designed the experiment. RT and MV provided the reagents. All authors contributed to the article and approved the submitted version.

## Conflict of Interest

The authors declare that the research was conducted in the absence of any commercial or financial relationships that could be construed as a potential conflict of interest.

## Publisher’s Note

All claims expressed in this article are solely those of the authors and do not necessarily represent those of their affiliated organizations, or those of the publisher, the editors and the reviewers. Any product that may be evaluated in this article, or claim that may be made by its manufacturer, is not guaranteed or endorsed by the publisher.

## Supplementary Material

Supplementary Figure 1 Actin depolymerizing agent Cytochalasin D (Cyto D) significantly affects HCMV entry into HIS cells. In this experiment, HIS cells either mock-treated **(A)** and or pre-treated with Cyto D (1.0 μg/ml: **B**) for 1 h before challenging with HCMV (10 MOI) for additional 2 h. The cells were then fixed and washed for immunofluorescence staining using anti-HCMV gB antibody for the virus detection using Nikon A1R confocal imaging at 40×. A high-affinity F-actin probe rhodamine phalloidin conjugated to red fluorescent dye was used for the detection of actin cytoskeleton, while DAPI was used for nucleus staining. The region boxed in **(A)** is further highlighted in (a–c) showing the presence of HCMV particles on the sprouting filopodial antennas.

Supplementary Figure 2 Enzymatic expression of 3-O-sulfotransferase-3 (3-OST-3) results in the entry of herpes simplex virus type-1 (HSV-1) into resistant CHO-K1 cells. CHO-K1 cells were grown in six-well plates to subconfluence and transfected with 2.0μg of human encoded 3-OST isoform (3-OST-3B1), or with an empty vector (pCDNA) using lipofectamine 2000. At 16 h post-transfection, the cells were replated into 96-well dishes for infection with recombinant virus HSV-1 (KOS) gL86 at the 10 MOI. After 6 h postinfection, β-galactosidase assays were performed using either a soluble substrate o-nitrophenyl-β-D-galactopyranoside (ONPG; ImmunoPure, Pierce) at 3.0 mg/ml. The enzymatic activity was measured at 410 nm using a microplate reader. Viral entry corresponds to expression of 3-O sulfated HS receptor. Asterisks indicate significant difference from controls and/or treatments (*n* = 3; *P* < 0.005, Student’s *t*-test) and error bars represent SD.

Supplementary Figure 3 The expression of HCMV glycoprotein B (gB) in CHO-K1 cell. In this experiment, CHO-K1 cells were transfected with the expression plasmid encoding HCMV gB and or an empty vector (pCDNA) using lipofectamine 2000 for 16 h. The cells were then washed with 1× Tris-buffered saline followed by methanol fixing. The gB detection was done using monoclonal antibody to HCMV gB and goat anti-mouse peroxidase conjugated secondary antibody followed by rinsing and adding the substrate 3,3′,5,5′-tetramethyl- benzidine (TMB). The enzymatic activity was measured at OD 450 nm by a microplate photometer (Thermo Fisher Scientific Multiskan FC). Asterisks indicate significant difference from controls and/or treatments (*n* = 3; *P* < 0.005, Student’s *t*-test) and error bars represent SD.
