# Supplementary figures and images for: Induction of Filopodia During Cytomegalovirus Entry Into Human Iris Stromal Cells
Source: Front Microbiol. 2022 Apr 5;13:834927. doi: 10.3389/fmicb.2022.834927 (PMC9018114; doi:10.3389/fmicb.2022.834927)

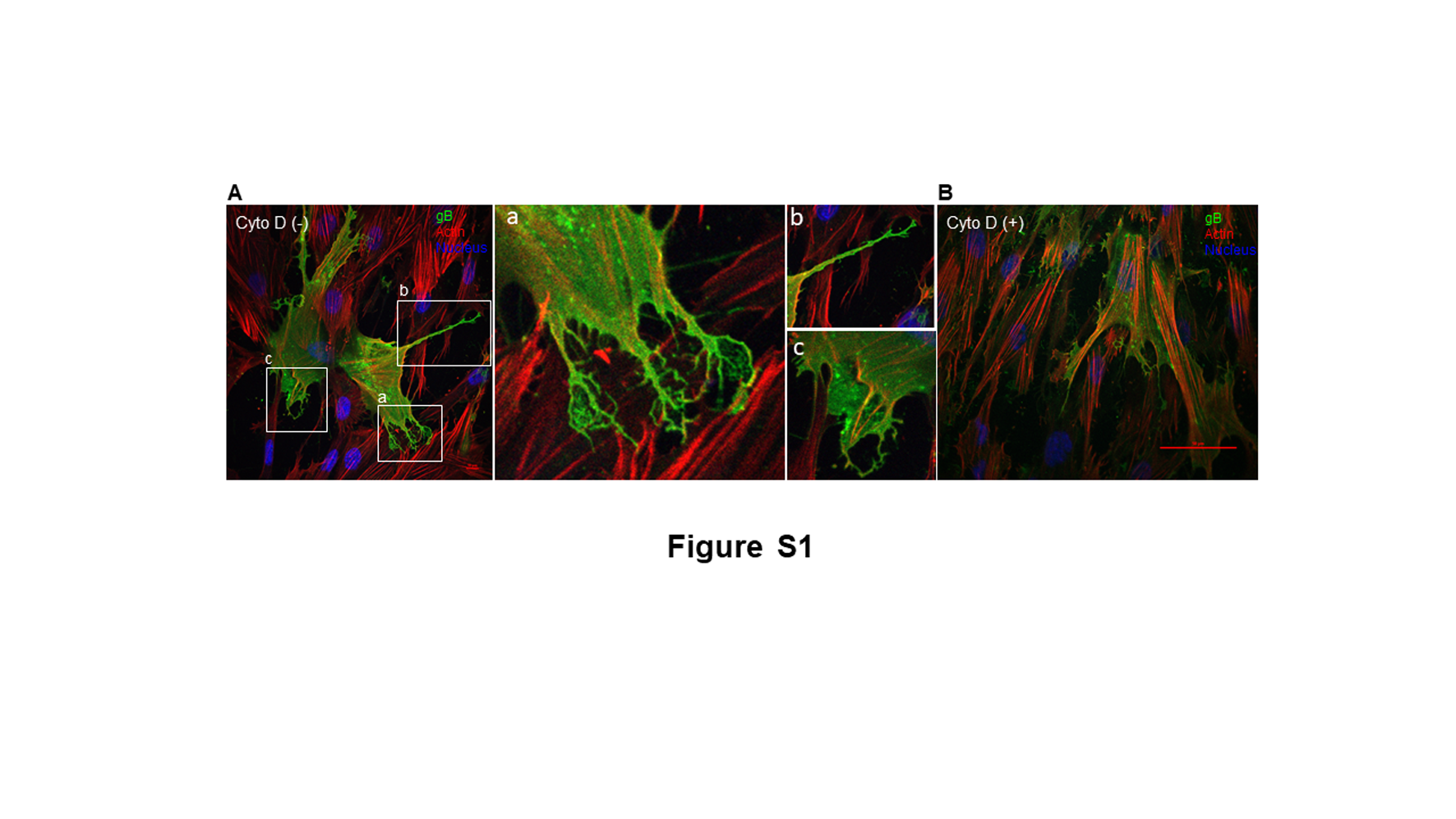

Supplement: Supplementary Figure 1 — Actin depolymerizing agent Cytochalasin D (Cyto D) significantly affects HCMV entry into HIS cells. In this experiment, HIS cells either mock-treated (A) and or pre-treated with Cyto D (1.0 μg/ml: B) for 1 h before challenging with HCMV (10 MOI) for additional 2 h. The cells were then fixed and washed for immunofluorescence staining using anti-HCMV gB antibody for the virus detection using Nikon A1R confocal imaging at 40×. A high-affinity F-actin probe rhodamine phalloidin conjugated to red fluorescent dye was used for the detection of actin cytoskeleton, while DAPI was used for nucleus staining. The region boxed in (A) is further highlighted in (a–c) showing the presence of HCMV particles on the sprouting filopodial antennas. [file Image_1.tif]

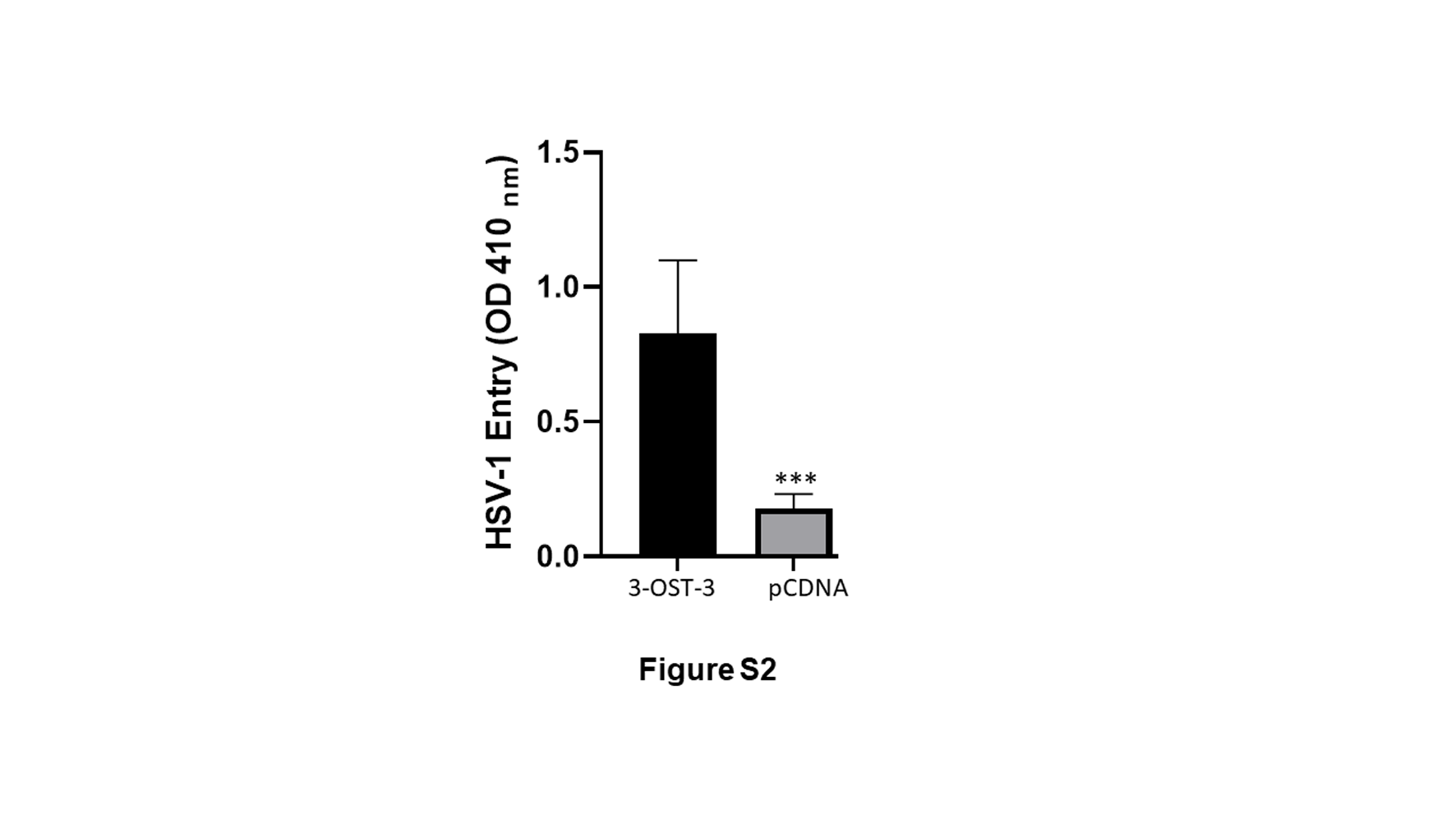

Supplement: Supplementary Figure 2 — Enzymatic expression of 3-O-sulfotransferase-3 (3-OST-3) results in the entry of herpes simplex virus type-1 (HSV-1) into resistant CHO-K1 cells. CHO-K1 cells were grown in six-well plates to subconfluence and transfected with 2.0μg of human encoded 3-OST isoform (3-OST-3B1), or with an empty vector (pCDNA) using lipofectamine 2000. At 16 h post-transfection, the cells were replated into 96-well dishes for infection with recombinant virus HSV-1 (KOS) gL86 at the 10 MOI. After 6 h postinfection, β-galactosidase assays were performed using either a soluble substrate o-nitrophenyl-β-D-galactopyranoside (ONPG; ImmunoPure, Pierce) at 3.0 mg/ml. The enzymatic activity was measured at 410 nm using a microplate reader. Viral entry corresponds to expression of 3-O sulfated HS receptor. Asterisks indicate significant difference from controls and/or treatments (n = 3; P < 0.005, Student’s t-test) and error bars represent SD. [file Image_2.tif]

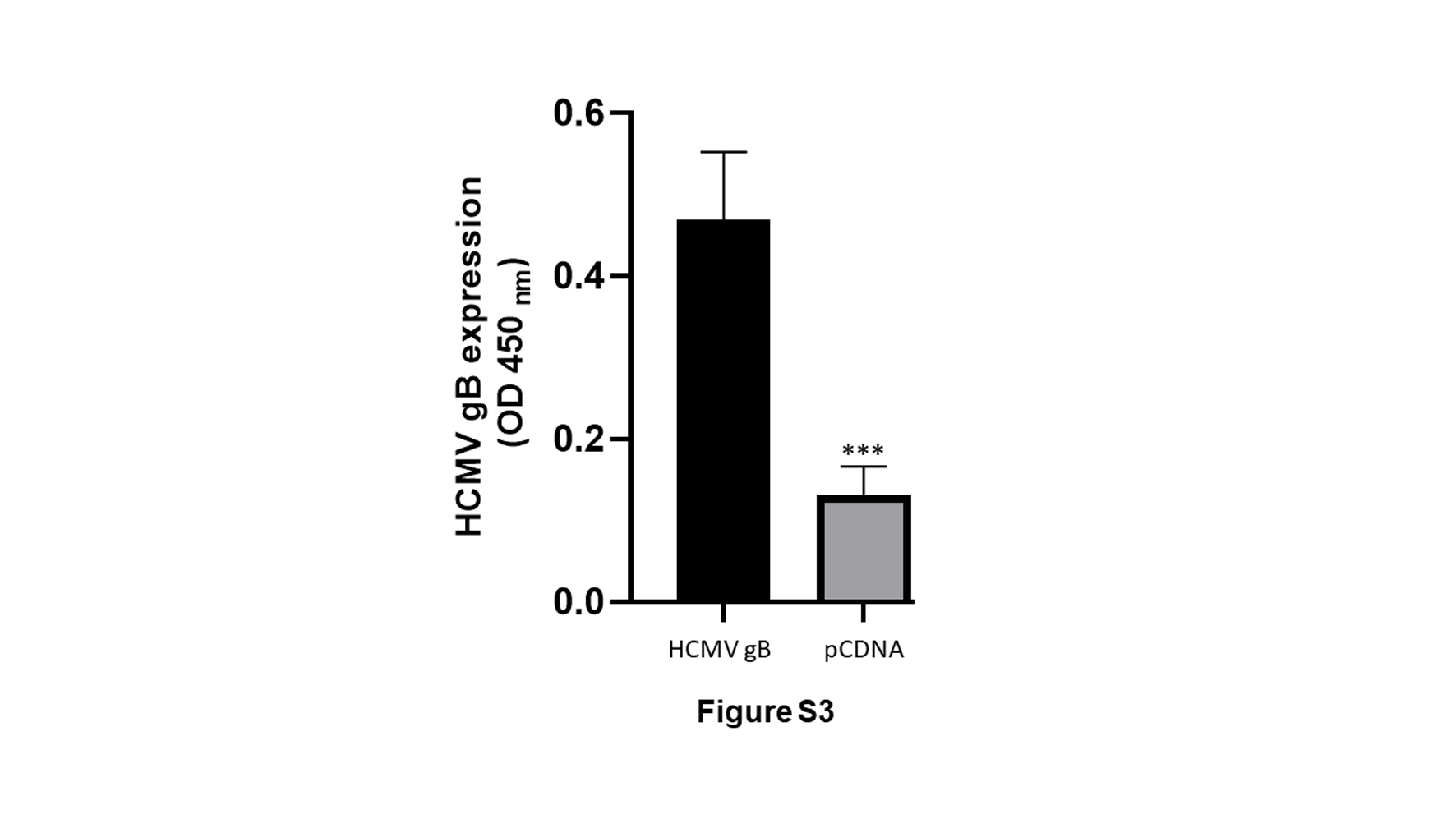

Supplement: Supplementary Figure 3 — The expression of HCMV glycoprotein B (gB) in CHO-K1 cell. In this experiment, CHO-K1 cells were transfected with the expression plasmid encoding HCMV gB and or an empty vector (pCDNA) using lipofectamine 2000 for 16 h. The cells were then washed with 1× Tris-buffered saline followed by methanol fixing. The gB detection was done using monoclonal antibody to HCMV gB and goat anti-mouse peroxidase conjugated secondary antibody followed by rinsing and adding the substrate 3,3′,5,5′-tetramethyl- benzidine (TMB). The enzymatic activity was measured at OD 450 nm by a microplate photometer (Thermo Fisher Scientific Multiskan FC). Asterisks indicate significant difference from controls and/or treatments (n = 3; P < 0.005, Student’s t-test) and error bars represent SD. [file Image_3.tif]
